# Determination of Dronedarone and Debutyldronedarone in Human Plasma by HPLC-UV

**DOI:** 10.3390/ijms26094304

**Published:** 2025-05-01

**Authors:** Paweł K. Kunicki, Adam Stocki

**Affiliations:** Department of Drug Chemistry, Pharmaceutical and Biomedical Analysis, Medical University of Warsaw, Banacha 1, 02-097 Warsaw, Poland; adam.stocki1999@gmail.com

**Keywords:** dronedarone, debutyldronedarone, HPLC-UV, bioanalysis

## Abstract

Dronedarone (DRO) is an antiarrhythmic drug that should be used under close supervision, and therapeutic drug monitoring (TDM) may be one of the tools supporting pharmacotherapy. The aim of our study was to develop an economical HPLC method for determining DRO and its active metabolite debutyldronedarone (DBD) in human plasma. An HPLC isocratic system with a manual injector was applied. The separation was performed on a Supelcosil LC-CN column (150 × 4.6 mm, 5 µm) at an ambient temperature. The mobile phase was a mixture of CH_3_OH:CH_3_CN:H_2_O:0.5 M KH_2_PO_4_ (170:85:237.2:7.8 (*v*/*v*)) + 0.1 mL 85% H_3_PO_4_ pumped at a flow rate of 1.8 mL/min. The UV detection was set at λ = 290 nm. A methyl tert-butyl ether was used for the extraction from a 0.4 mL alkalized plasma sample. The analytes were eluted at retention times of 4.0 min, 5.2 min and 6.0 min for DBD, internal standard bepridil and DRO, respectively. The method was calibrated in the range of 10–1000 ng/mL for both DRO and DBD. The adequate specificity, accuracy and precision were demonstrated in accordance with EMA guidelines, i.e., ≤15% (≤20% for the LLOQ), which ensures the reliability of the measurements. This method can be recommended for laboratories with basic HPLC equipment for TDM, adherence assessments and even in PK studies during chronic DRO therapy.

## 1. Introduction

Dronedarone (DRO) is a relatively new (FDA, EMA-approved 2009) antiarrhythmic drug that was developed as a safer alternative to amiodarone (AD), a structurally similar benzofuran derivative. The removal of two iodine atoms from the molecule was expected to have a beneficial effect on the drug’s safety profile, particularly in terms of thyroid dysregulation and pulmonary toxicity [1,2]. Clinical trials showed a beneficial antiarrhythmic effect in terms of atrial fibrillation compared to a placebo [2,3,4,5]. The promising future of DRO has been verified by clinical studies, which have proven that, in comparison to AD, it is a less effective drug, although it is safer to use [3,6,7]. Currently, DRO is indicated to reduce the risk of hospitalization for atrial fibrillation in a selected group of patients in sinus rhythm with a history of paroxysmal or persistent atrial fibrillation [3,4,5,8,9,10]. The drug should be administered with a high-fat meal, which provides a several times (15% vs. 4%) higher bioavailability than after dosing on an empty stomach [5]. The administration of DRO inconsistent with these recommendations may reduce the clinical effectiveness of the therapy [11]. Dronedarone is extensively metabolized (CYP3A4), and the primary metabolite is debutyldronedarone (DBD) which is pharmacodynamically active but is one-tenth to one-third as potent as DRO [5,6]. At the same time, the use of DRO should be carefully supervised, and one of the tools supporting the optimization of pharmacotherapy may be therapeutic drug monitoring (TDM) and adherence control. Their introduction requires access to appropriate methods for determining the plasma drug concentration in patients treated chronically with DRO. There are only few published methods for the chromatographic determination of DRO (or DRO plus DBD) in biological material that can be routinely used in plasma/serum [12,13,14,15,16,17,18]. Among them, analytical procedures using the LC-MS/MS technique were developed for pharmacokinetic studies after a single dose administration of DRO in volunteers [15,16] and in an animal model [17,18]. They are therefore characterized by a suitably low limit of quantification (LLOQ) for DRO, between 0.1 and 1 ng/mL, but only the publication by Baek et al. provides a measurement range wide enough (1–1000 ng/mL) for analyses during the chronic use of DRO (drug accumulation) [17]. On the other hand, the disadvantage of each of the three remaining publications based on HPLC with UV detection is the range of the method, which does not allow for a reliable determination of low drug concentrations [12,13,14].

The aim of our study was to develop a simple and economical HPLC method for determining DRO and its active metabolite DBD in human plasma for use in TDM and to assess patient adherence as well as for pharmacokinetic studies conducted at a steady state.

## 2. Results

### 2.1. Method Development

#### 2.1.1. Chromatographic Separation

For the chromatographic separation, we used a cyanopropyl derivatized silica phase (LC-CN) column, which has been useful in many of our bioanalytical applications. For this type of packing, the appropriate mobile phase is based on an acetonitrile–water mixture with a moderate acidification. The optimal acetonitrile content, as well as the phosphate concentration in the mobile phase, was selected experimentally. When using the initial mobile phase composition of CH_3_OH:CH_3_CN:H_2_O:0.5 M KH_2_PO_4_ (200:100:194:6 *v*/*v* + 0.1 mL 85% H_3_PO_4_), at a flow rate of 1.5 mL/min, there was an acceptable separation of the DBD–DRO peaks at a retention time (RT) of 3.3 and 4.2 min, respectively. Unfortunately, both considered internal standards (ISs), i.e., bepridil (BEP) and tamoxifen (TAM) were co-eluted with DRO at a RT of 4.0–4.2 min. Interestingly, both compounds tested as ISs showed similar elution characteristics. To effectively improve the separation of all compounds, the following were checked: (1) the optimal proportion of organic components in the mobile phase (methanol–acetonitrile), (2) the effect of the polarity of the mobile phase (% of methanol–acetonitrile content) and (3) the effect of the ionic strength of the mobile phase (0.5 M KH_2_PO_4_ content). In the first test (1), no significant impact of changing the CH_3_OH-CH_3_CN ratio on the separation was confirmed, and the optimal ratio was left as 2:1, *v*/*v*. Decreasing the % of organic solvents balanced with water (test 2) resulted in an increase in RTs and allowed the peaks to move further apart. The compounds of BEP/TAM and DRO were completely separated with a retention time difference of 0.9 min for the following mixture: CH_3_OH:CH_3_CN:H_2_O:0.5M KH_2_PO_4_ (170:85:238:7 *v*/*v* + 0.1 mL 85% H_3_PO_4_). It was found (test 3) that a larger amount of 0.5 M KH_2_PO_4_ proportionally shortens the elution time of all compounds and is therefore not decisive for their separation. To shorten the run-time, a mobile phase containing 12 mL of 0.5 M KH_2_PO_4_ was selected. During further studies with human plasma an interference was, however, observed with the matrix components eluting at a time similar to the retention time of DBD. Since reducing the ionic strength of the mobile phase effectively increased the retention time of the analytes relative to the endogenous interferences, a mobile phase composition suitable for the analysis in plasma was finally established: CH_3_OH:CH_3_CN:H_2_O:0.5 M KH_2_PO_4_ (170:85:237.2:7.8 *v*/*v* + 0.1 mL 85% H_3_PO_4_). Aiming for a short run-time, the flow has been increased to 1.8 mL/min.

It was decided that BEP would be used as the IS, as it is a drug currently not approved for treatment in the EU and the US, which means that this substance (unlike TAM) will not be found in the biological material obtained from patients. Under the analytical conditions presented, the LC-CN column provided the complete separation of DRO, DBD and BEP (IS) from each other and from the matrix constituents.

#### 2.1.2. Extraction

**Figure 1:**
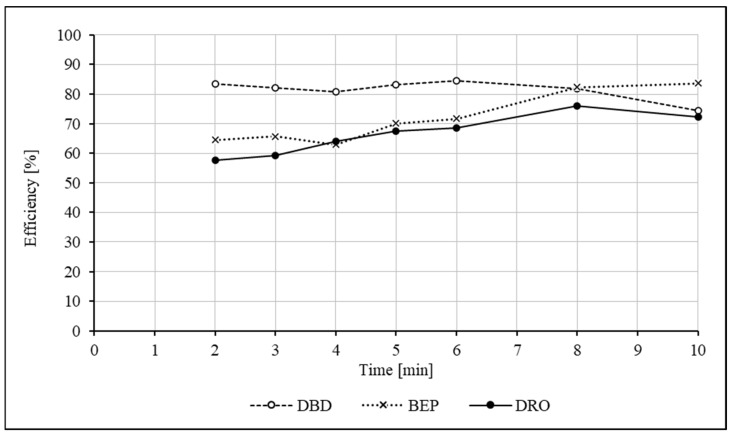
The influence of the mixing time using a rotary mixer on the extraction efficiency.

For the isolation of analytes from the biological matrix, the standard liquid–liquid extraction was chosen. The effectiveness of five selected solvents was tested: diisopropyl ether, methyl tert-butyl ether (MTBE), ethyl acetate, hexane and dichloromethane. Ethyl acetate, despite its relatively low biological background, yielded only about 50%. Diisopropyl ether, on the contrary, caused a very high biological background interfering with the signals of the analytes. Hexane did not provide a satisfactory extraction for DBD, while dichloromethane was ineffective for DRO and BEP. The most promising of the above-mentioned solvents turned out to be MTBE, due to the high efficiency of 70–80% and the relatively high chromatographic purity of the sample. The most favorable environment for extraction was provided by the alkalization of the plasma sample to a pH = 11.5–11.8 by adding 50 µL of a 10% Na_2_CO_3_ solution. The efficiency of DRO was higher by approximately 7% when the extraction was performed at a slightly acidic pH, but this was at the cost of a significant reduction in efficiency to 65% and 62% for DBD and BEP, respectively. The volume of the extraction reagent was experimentally optimized to 3 mL, and this volume was used for the method validation. To evaluate the effect of the extraction time on its efficiency, the samples were extracted for 2, 3, 4, 5, 6, 8 and 10 min using 4 mL of MTBE. The results are presented in the graph (Figure 1).

The highest DRO recovery was obtained when the process was carried out for 8 min; DBD is characterized by a relatively constant efficiency in the range up to 8 min. Unexpectedly, the DRO and DBD efficiency decreased for the 10 min measurement. We found that in the case of BEP, the efficiency increases with the extraction time. It was finally decided that the optimal time for extraction would be 8 min. The absolute recovery was analyzed by comparing the peak areas for the extracted calibration standards with those obtained from the direct injection of equivalent quantities of standards with regard to the volumetric relations. The extraction efficiencies [%] for samples tested during the intra- and inter-assay accuracy and precision studies were as follows (n = 39): 79.0% (73.4–84.9%) for DRO, 78.8% (69.9–81.8%) for DBD and 79.7% for BEP.

#### 2.1.3. Analytical Wavelength

The spectrophotometric detection was calibrated at λ = 290 nm, where there is a second absorption maximum for DRO and DBD with a signal intensity of ~44% compared to the main maximum occurring at λ = 216 nm. The analytical wavelength of 290 nm was chosen because a massive interference from the biological matrix was observed at 216 nm.

### 2.2. Method Validation

#### 2.2.1. Specificity

**Figure 2:**
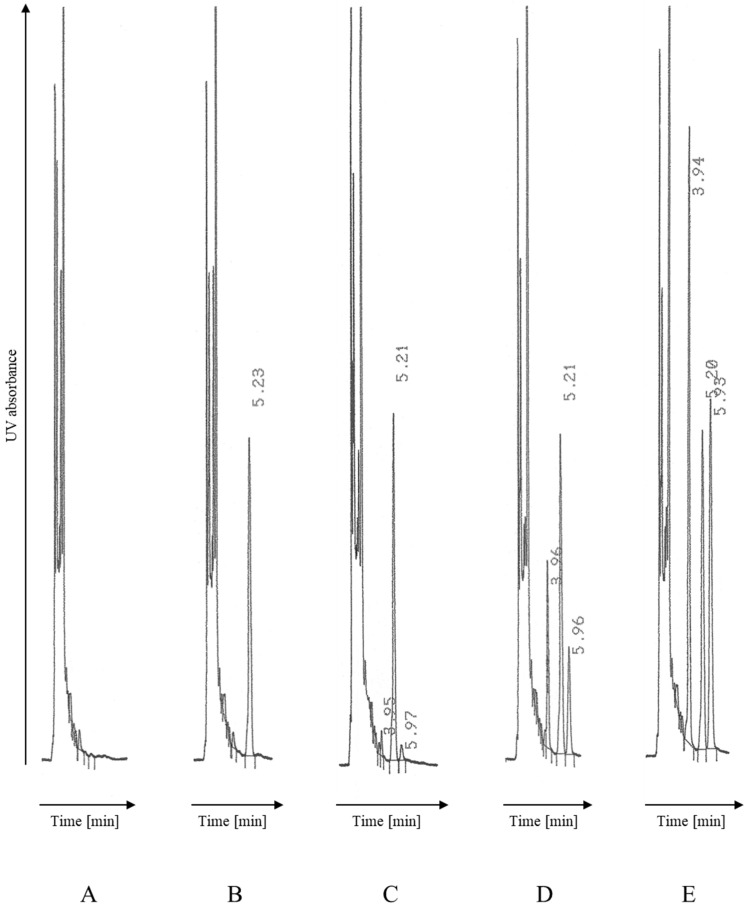
Chromatograms of extracted plasma samples analyzed as described in Materials and Methods (signal attenuation 16): (**A**) drug-free plasma analyzed without IS; (**B**) drug-free plasma analyzed with IS; and (**C**–**E**) drug-free plasma spiked with DRO/DBD to obtain concentration of (**C**) 30 ng/mL (QC-L), (**D**) 250 ng/mL (QC-M) and (**E**) 800 ng/mL (QC-H). Peaks: DBD: 3.9–4.0 min, BEP (IS): 5.2 min and DRO: 5.9–6.0 min.

As previously reported, the analytes were well separated from each other, showing symmetrical peaks without a significant tailing effect at 4.0 min, 5.2 min and 6.0 min for DBD, BEP and DRO, respectively (Figure 2). Twelve plasma samples (the surplus material collected for routine laboratory tests) from random anonymous cardiac patients not treated with DRO were analyzed and evaluated for interference. In the samples after extraction, the signal of the endogenous compound was observed with a retention time similar to that of BEP and DRO. An additional centrifugation step allowed us to greatly reduce the interference from the plasma components, and no significant interference was observed under the final chromatographic conditions.

#### 2.2.2. Calibration and Linearity

To evaluate the linearity of the detection system response, the prepared calibration and control solutions were applied in triplicate onto the column at a constant volume = 15.6 μL, which corresponded to the plasma DRO and DBD concentration levels in the range of 10–1000 ng/mL. The detector response for the analytes was easily described by linear equations:

DRO: y = 347.36 x − 1646.8, r^2^ = 0.9999, (1)

DBD: y = 374.34 x − 1213.3, r^2^ = 0.9999, (2)

Having confirmed the response from the UV detector, the method was calibrated and found to be linear up to the upper limit of quantitation (ULOQ). The calibration curves were obtained by analyzing spiked plasma samples for each of the six concentrations tested, i.e., 10–40–100–200–500–1000 ng/mL in duplicates. It was not necessary to weight the data for the method calibration. The curves for DRO and DBD were described by the following equations:

F_DRO_ = 0.0014659 DRO + 0.0031, r^2^ = 0.9966, (3)

F_DBD_ = 0.0016640 DBD + 0.0047, r^2^ = 0.9922, (4)

where DRO and DBD stand for the respective concentration in ng/mL, and F_DRO, DBD_ are factors (ratios) obtained from the peak areas: DRO or DBD/IS.

#### 2.2.3. Precision and Accuracy

To assess the precision and accuracy of the method, DRO and DBD were measured at five concentration levels, i.e., the LLOQ (quality controls (QCs): low (QC-L), medium (QC-M) and high (QC-H) and ULOQ as described in Section 4.4. The intra-assay (within-run) evaluation was performed four times for each level. The inter-assay (between-run) testing was composed of five independent runs, including four runs with a single sample and one quadruplicate run, which was serving for the intra-assay. The precision for the DRO determination was calculated at an RSD of 2.4–11.0% for the intra-assay and 2.1–13.7% for the inter-assay. The accuracy for DRO was found to be between 87.5% and 105.4% for the intra-assay and between 98.1% and 105.1% for the inter-assay. The precision for DBD yielded 3.8–17.3% (at the LLOQ) for the intra-assay and 2.8–13.8% for the inter-assay. The accuracy for DBD was found to be between 87.8% and 108.2% for the intra-assay and between 93.1% and 110.2% for the inter-assay. The detailed results are shown in Table 1. The results fulfilled both the EMA and FDA requirements regarding method precision and accuracy [19,20].

#### 2.2.4. Limit of Quantification, Range, Dilution Integrity and Carry-Over

The lower limit of quantification (LLOQ) was the lowest calibration standard with an acceptable accuracy and precision (Table 1). The LLOQ for DRO as well as for DBD was set at 10 ng/mL. The method was calibrated in the range of 10–1000 ng/mL (LLOQ—ULOQ) for each analyte. The dilution integrity parameter was not determined because the ULOQ value was set at such a high level that the possibility of exceeding it in a patient plasma sample is extremely unlikely. The carry-over effect was detected by injecting extracted blank samples (drug-free) after the ULOQ sample. No carry-over effect was observed.

## 3. Discussion

As planned, we developed a method to monitor the concentration of DRO and its metabolite DBD in human plasma. We used a simple, isocratic HPLC-UV technique, assuming that it would be sufficient for our purposes and that the values of the expected concentrations do not require the use of chromatography with sensitive mass detection (LC-MS/MS). In all methods published for DRO, their authors applied columns with C18 packing [12,13,14,15,16,17,18]. Based on many years of experience in determining the structurally very similar amiodarone and its metabolite desethylamiodarone (DEAD) [21], we used a well-proven column with -CN (cyanopropyl derivatized silica phase) packing. The composition of the mobile phase was experimentally optimized to obtain a separation with an appropriate selectivity. Considering the application of the method to samples from patients treated with antiarrhythmic drugs, we deliberately refrained from using AD and/or DEAD as an internal standard (contrary to the publications by Baek and Bolderman et al. [12,17]). We assumed that these compounds (AD and DEAD) may be present in the plasma of some patients treated with DRO at the time of sampling and treated with extremely slowly eliminated AD in the preceding period of several months. We experimentally determined that tamoxifen and bepridil can serve as ISs, and the latter was finally selected due to its absence from the list of compounds approved for treatment in both the EU and the US. For the isolation of analytes from plasma, a simple single liquid–liquid extraction with MTBE was used after a prior plasma alkalization to a pH = 11.5–11.8. For the established analytical conditions, the selective separation of compounds was obtained avoiding interference with components of the biological matrix. The time of the chromatographic analysis was relatively short, amounting to about 7.5 min. The measurement ranges (10–1000 ng/mL for each analyte) were confirmed in accuracy and precision studies, meeting the criteria of the EMA and FDA guidelines [19,20], i.e., inaccuracy and precision < 15% (<20% for LLOQ), which proves the measurement reliability. Recently, the results of the clinical trials EURIDIS and ADONIS, which were conducted much earlier, have been published by Thind et al. in the part relating to the pharmacokinetics of DRO [9]. They presented data for 1795 samples collected from 507 patients who were given the commonly recommended DRO dose of 400 mg bid over a period of one year. The evaluation of steady-state trough concentration values clearly indicates that the analytical method for monitoring DRO in humans should have a ULOQ no lower than 400 ng/mL [9]. In the case of published LC-MS/MS methods, this condition is met only for the Baek method applied for the determination of DRO in rat plasma (ULOQ = 1000 ng/mL) [17]; the others having a ULOQ for DRO between 100 and 200 ng/mL require an extension of the measurement range [15,16,18]. At this point we would like to refer to the available HPLC-UV methods. The method range proposed for plasma (40–5000 ng/mL) by Boldermann et al. is too shifted towards high concentrations, and the multi-step analytical procedure is complicated [12]. Bin Jardan et al. describe the determination of DRO alone in rat plasma with a LLOQ of only 25 ng/mL. Despite the small sample volume (0.1 mL), this assay is characterized by a long run-time (20 min) and a complicated sample preparation procedure [13]. The method presented by Chadha et al. is calibrated and validated for an inappropriate concentration range (2–50 µg/mL), which makes it impossible to use in patient samples [14]. Our method has the lowest LLOQ (10 ng/mL) among the HPLC-UV methods and is therefore the only one that offers a real application possibility for determinations in plasma samples from patients. Some readers may consider the lack of the clinical application of the presented procedure as a limitation. It is worth noting, however, that none of the methods published so far have been used for TDM, and those used to measure DRO and DBD concentrations in patients (LC-MS/MS method with a LLOQ = 0.5 ng/mL) have not been published [9,11]. DRO and DBD are chemical compounds with a proven, sufficient stability in human plasma under various conditions and storage times, which was clearly documented by Xie et al. and Parekh et al. for short- and long-term stability as well as in the freeze–thaw tests [15,16]. Our method showed an accuracy and precision sufficient for a reliable bioanalytical method and an adequate specificity according to the EMA guideline [19]. An undoubted advantage is the use of a simple, isocratic HPLC-UV system, which favors its availability in laboratory practice. Potential modifications by replacing the manual injector with an autosampler and the integrator with software may further improve its attractiveness and ease of use. A significant percentage of ineffective antiarrhythmic therapy with dronedarone may be related to subtherapeutic dosing, the incorrect administration of the drug on an empty stomach or generally a lack of adherence to therapy. One way to monitor adherence is to control the concentration of drugs in the patient’s blood, which requires analytical tools in a bioanalytical laboratory. The presented reliable and economical method for determining the concentration of DRO and its active metabolite DBD in human plasma may serve such an optimization of pharmacotherapy.

## 4. Materials and Methods

### 4.1. Chemicals

**Figure 3:**
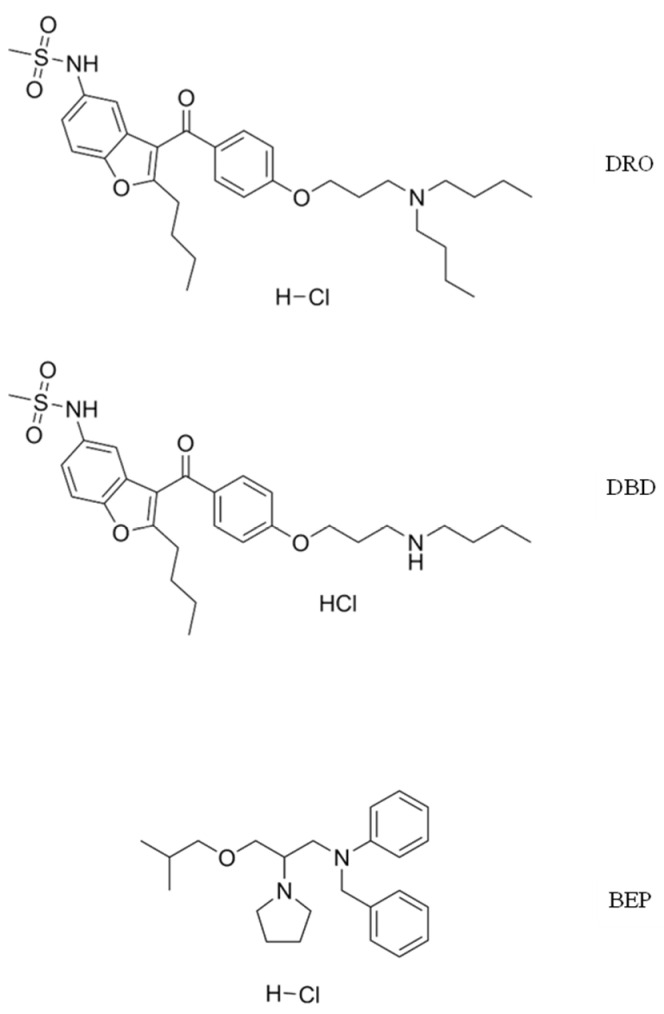
The chemical structures of dronedarone (DRO), debutyldronedarone (DBD) and the internal standard bepridil (BEP).

Dronedarone (DRO) hydrochloride and debutyldronedarone (DBD) hydrochloride were from MedChemExpress (Monmouth Junction, NJ, USA); bepridil (BEP) hydrochloride and tamoxifen (TAM) were obtained from Sigma-Aldrich (Steinheim, Germany). The chemical structures are shown in Figure 3. HPLC-grade methanol, dichloromethane, n-hexane and 85% H_3_PO_4_ were from J.T. Baker (Deventer, Netherlands). HPLC-grade acetonitrile, diisopropyl ether, KH_2_PO_4_ (pro analysi) and Na_2_CO_3_ (pro analysi) were from Merck (Darmstadt, Germany). HPLC-grade methyl tert-butyl ether (MTBE) was from Chempur (Piekary Śląskie, Poland). HCl was from POCH S.A. (Gliwice, Poland) and HPLC-grade ethyl acetate was from Sigma-Aldrich (Steinheim, Germany). HPLC-grade water was obtained from a water deionizer.

### 4.2. Instrumentation

The HPLC isocratic system (Spectra-Physics, San Jose, CA, USA) consisted of a P100 pump, a Spectra 100 UV detector, an injector with 50 µL loop (Model 7125i, Rheodyne, Cotati, CA, USA) and a Chrom Jet 4400 integrator. Universal laboratory centrifuges, MPW 375 and MPW-260R (MPW, Warsaw, Poland), a vortex-shaker Reax top and a rotary mixer Reax 2 (Heidolph Instruments, Schwabach, Germany), a water bath (LW 502, AJL Electronic, Krakow, Poland), an ultrasonic cleaner PS-10A (CNC-Ultrasonic, Jedlnia Letnisko, Poland), a universal laboratory pump KL 1-02 (AGA LABOR, Warsaw, Polska) and a water deionizer DL3-100 (Polwater, Krakow, Poland) were used for sample preparation and supporting laboratory activities.

### 4.3. Chromatographic Conditions

The separation of analytes was performed on Supelcosil LC-CN column (150 × 4.6 mm, 5 µm) protected with Supelguard LC-CN precolumn (20 × 4.6 mm, 5 µm), both from Supelco, (Bellefonte, PA, USA). The mobile phase was a mixture of CH_3_OH:CH_3_CN:H_2_O:0.5 M KH_2_PO_4_ (170:85:237.2:7.8 (*v*/*v*)) + 0.1 mL 85% H_3-_PO_4_). The isocratic flow rate was 1.8 mL/min with operating pressure of approx. 120 bar. All analyses were performed at ambient temperature. UV detection was set at a wavelength of 290 nm.

### 4.4. Stock and Working Solutions, Calibration and Quality Controls

Stock solutions of DRO, DBD, BEP and TAM (1 mg/mL) were prepared by weighing and dissolving appropriate amounts of their chemically pure hydrochlorides (chemically pure substance for TAM) in methanol and then stored tightly closed at 4 °C. The working solutions for calibration and controls were prepared from the stock solution by adequately diluting in methanol. Working solutions were added to drug-free plasma to obtain DRO as well as DBD concentration levels of 10–40–100–200–500–1000 ng/mL (calibration) and of 30–250–800 ng/mL (quality controls: QC-L, QC-M and QC-H, respectively). Internal standard (BEP) working solution (80 µg/mL) was prepared from the stock solution by adequate dilution in methanol. All working solutions were then stored refrigerated at 4 °C. Stock and working solutions were stable for 8 weeks of storage.

### 4.5. Sample Preparation

Drug-free plasma necessary for method development and validation was obtained from a blood bank: Regional Blood Donation and Blood Treatment Center in Warsaw, Poland.

A total of 400 µL of plasma was transferred into a 15 mL Pyrex^®^ screw cap glass tube, first spiked with 25 µL of calibration/QC working solution (or methanol for patient sample) and vortexed for 10 s, next spiked with 20 µL of IS working solution and vortexed for 10 s; then, 50 µL of 10% Na_2_CO_3_ solution was added and the sample was vortexed for 10 s. Then, 3 mL of MTBE was added, and the sample was extracted for 8 min using rotary mixer. After centrifugation (2000× *g*, 10 min, ambient temperature) and freezing at −30 °C for 1 h, MTBE was transferred quantitatively into a 10 mL Pyrex^®^ conical glass tube and further evaporated to dryness in a water bath at 37 °C under a stream of argon. Then, the dried extract was reconstituted in 80 µL of a mobile phase, centrifuged (6000× *g*, 10 min, ambient temperature) and a 50 µL supernatant sample was carefully drawn and injected onto the column.

### 4.6. Method Validation

The method was validated in accordance with the current European Medicines Agency (EMA) guideline ICH M10 for bioanalytical methods [19]. The following parameters were determined: specificity, calibration, linearity, range with LLOQ, accuracy and precision. The EMA guideline defines the degree of closeness of the measured value to the nominal value as accuracy (not *trueness*). Following EMA, we calculated the accuracy as follows:

Accuracy (%) = (Measured Value/Nominal Value) × 100.

(Since we presented the accuracy result (Table 1) as the difference from 100%, we used the term inaccuracy, where inaccuracy = accuracy—100%.)

Precision is presented as the relative standard deviation (RSD) expressed as a percentage: RSD (%) = (Standard Deviation/Mean) × 100 [19]. Details for determining other parameters are described in Section 2.2 Method Validation.

## 5. Conclusions

The developed method for the simultaneous determination of DRO and DBD in human plasma can be used for TDM, adherence assessments and even in pharmacokinetic studies during chronic dronedarone therapy. This method can be recommended for laboratories equipped with basic HPLC equipment as an economical alternative to the LC-MS/MS technique. The method was validated using spiked human plasma, and the clinical verification using samples from patients treated with DRO is indicated before implementations in routine practice.

## Figures and Tables

**Table 1:** Accuracy and precision (n = 4).

		INTRA-ASSAY			INTER-ASSAY		
Analyte	Level/Concentration Added [ng/mL]	Concentration Determined (mean ± SD) [ng/mL]	Precision (RSD) [%]	Inaccuracy [%]	Concentration Determined (mean ± SD) [ng/mL]	Precision (RSD) [%]	Inaccuracy [%]
DRO	LLOQ—10	8.8 ± 1.0	11.0	−12.5	10.5 ± 1.4	13.7	+4.5
	QC-L—30	31.3 ± 3.3	10.6	+4.4	31.1 ± 1.5	4.9	+3.8
	QC-M—250	246.4 ± 5.8	2.4	−1.4	245.4 ± 5.2	2.1	−1.9
	QC-H—800	824.3 ± 54.6 *	6.6 *	+3.0 *	812.6 ± 47.1	5.8	+1.6
	ULOQ—1000	1054.4 ± 25.2	2.4	+5.4	1051.2 ± 22.8	2.2	+5.1
DBD	LLOQ—10	8.8 ± 1.5	17.3	−12.2	9.3 ± 1.3	13.8	−6.9
	QC-L—30	30.7 ± 3.2	10.3	+2.3	30.2 ± 3.3	11.0	+0.8
	QC-M—250	254.0 ± 13.5	5.3	+1.6	254.2 ± 14.3	5.6	+1.7
	QC-H—800	865.7 ± 32.8 *	3.8 *	+8.2 *	881.7 ± 24.6	2.8	+10.2
	ULOQ—1000	1042.8 ± 47.8	4.6	+4.3	1039.9 ± 43.3	4.2	+4.0

Inaccuracy = accuracy—100%. * n = 3.

## Data Availability

The original contributions presented in the study are included in the article, further inquiries can be directed to the corresponding author.

## Abbreviations

The following abbreviations are used in this manuscript:

HPLC	high-performance liquid chromatography
UV	ultraviolet
LC-MS/MS	liquid chromatography–tandem mass spectrometry
LC-CN	cyanopropyl derivatized silica phase
EMA	European Medicines Agency
FDA	Food and Drug Administration
C18	octadecyl column
CYP	cytochrome P450
PK	pharmacokinetic
